# Comparisons between small ribosomal RNA and theoretical minimal RNA ring secondary structures confirm phylogenetic and structural accretion histories

**DOI:** 10.1038/s41598-020-64627-8

**Published:** 2020-05-06

**Authors:** Jacques Demongeot, Hervé Seligmann

**Affiliations:** 1 Université Grenoble Alpes, Faculty of Medicine, Laboratory AGEIS EA 7407, Team Tools for e-Gnosis Medical & Labcom CNRS/UGA/OrangeLabs Telecoms4Health, F-38700 La Tronche, France; 2 The National Natural History Collections, The Hebrew University of Jerusalem, 91404 Jerusalem, Israel

**Keywords:** Evolution, Computational models

## Abstract

Ribosomal RNAs are complex structures that presumably evolved by tRNA accretions. Statistical properties of tRNA secondary structures correlate with genetic code integration orders of their cognate amino acids. Ribosomal RNA secondary structures resemble those of tRNAs with recent cognates. Hence, rRNAs presumably evolved from ancestral tRNAs. Here, analyses compare secondary structure subcomponents of small ribosomal RNA subunits with secondary structures of theoretical minimal RNA rings, presumed proto-tRNAs. Two independent methods determined different accretion orders of rRNA structural subelements: (a) classical comparative homology and phylogenetic reconstruction, and (b) a structural hypothesis assuming an inverted onion ring growth where the three-dimensional ribosome’s core is most ancient and peripheral elements most recent. Comparisons between (a) and (b) accretions orders with RNA ring secondary structure scales show that recent rRNA subelements are: 1. more like RNA rings with recent cognates, indicating ongoing coevolution between tRNA and rRNA secondary structures; 2. less similar to theoretical minimal RNA rings with ancient cognates. Our method fits (a) and (b) in all examined organisms, more with (a) than (b). Results stress the need to integrate independent methods. Theoretical minimal RNA rings are potential evolutionary references for any sequence-based evolutionary analyses, independent of the focal data from that study.

## Introduction

Ribosomes presumably evolved through serial accretions of tRNAs and tRNA-like RNAs^1–11^. The ribosomal dimeric RNA core surrounding the peptide synthesis site^12–14^ also resembles tRNA dimers linked by complementary anticodons, according to the self-referential hypothesis on the origin of translation^15–19^. Evidence for this process exists also in modern vertebrate mitochondrial ribosomes: regular mitochondrial tRNAs constitutively fulfill 5S rRNA functions^20,21^. In the latter case, extreme mitogenome reduction perhaps reversed evolution to a tRNA-insertion stage, enabling further mitogenome reduction. These evidences suggest that rRNAs derived from tRNAs.

## tRNA accretion

Several hypotheses suggest different historical scenarios for tRNA evolution, all assuming accretions of smaller sequences^22–41^. A similar hypothesis exists for 5S rRNAs^42^.

Some evidence suggests that tRNAs originate from stem-loop hairpins initiating replication^43–50^. Other analyses show striking similarities in nucleotide triplet biases of tRNAs and protein coding genes^51,52^. Theoretical RNA rings, sequences artificially designed according to coding constraints^53,54^ seem homologous to tRNAs^55–57^.

## rRNA accretion history: cladistics

Two main approaches have been developed and used to recover accretion histories of ribosomal RNAs. Both consider secondary structure subcomponents of rRNAs as units undergoing this process. One approach is based on homology, character polarity^58^ and cladistic comparisons to infer accretion history from comparisons among numerous sequences^59^. This classical comparative biology method uses parsimony as its main conceptual tool^60^ and was also used to recover evolution of molecular functions^61,62^ and protein accretion^63,64^. Various empirical tests show that this method recovers actual histories better than chance^65–75^.

## rRNA accretion history: structure

A second approach is structure-based, and assumes that the ribosome grew from its spatial core towards its periphery, with the most ancient structural subcomponents located at the physical center of the ribosome, and the more recent ones at its periphery^76–78^. The method corresponds to that of spatial comparisons in disciplines such as plant community ecology. Structures encompass large amounts of information: in ribosomes, contact biases between amino acids and nucleotide triplets recover the very ancient evolution of genetic code codon-amino acid assignments^79^. Though reasonable, the structural method lacks to our knowledge further empirical tests in contexts of reconstructing biomolecular histories, but one of its merits is that for each taxon for which accurate structural data are available, it produces (slightly) different histories, enabling to search for consensuses.

The theoretical premises of the structural approach are in observations that ontogenies of different structures recover their phylogenies: chemical prebiotic evolution^80^; genetic code evolution^81^; embryology^82,83^; and ecological communities^84^. Spatial variation in vegetation can reconstruct the ontogeny of forests (forest succession^85^), but plant colonization at forest periphery and clearings differ from *de novo* colonization of areas where no forest is adjacent and no humus exists: primary and secondary successions differ^86^. In addition, the structural model unrealistically assumes equal ribosomal growth in all directions from the core to the periphery^87–89^. Its name, the onion peeling model, is formally incorrect (in onions, peripheral rings are most ancient), reflecting emphasis on structure rather than historical process^90^.

### Comparing accretion histories: cladistic vs structure

**Figure 1:**
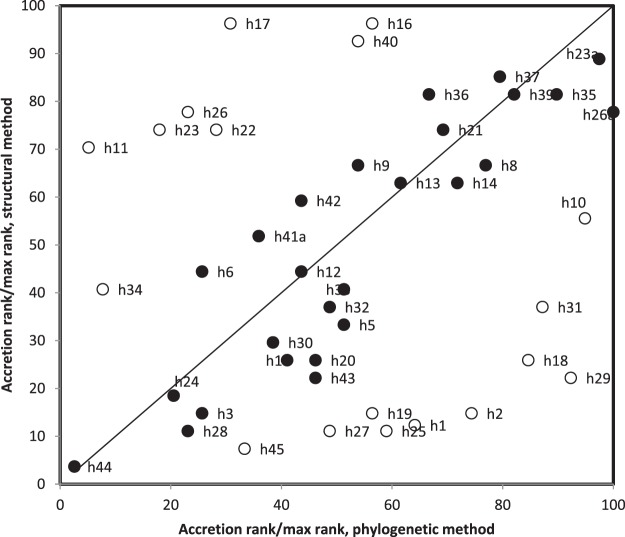
Accretion rank of 16S rRNA structural subelements according to the structural onion model (periphery most recent^78^ ranks therein from Fig. 2) as a function of accretion rank according to the phylogenetic method (^59^, ranks are therein from the phylogenies for 16S secondary structure elements in the Fig. 2 and in their corresponding supplementary figure). Accretion ranks are divided by the highest rank according to that method (structural, 27; phylogeny, 39), then multiplied by 100. Full symbols indicate structural subelements for which the absolute value of the difference between accretion ranks (divided by maximal ranks) is <25, hollow symbols have differences >25. Considering all 44 datapoints, the correlation between the two methods is r = 0.308, P = 0.021, meaning that 9.5% of the variation is common between methods (**a,b**); for the 26 filled symbols, r = 0.898, P = 0, 80.6% of the variation is common. Hence methods (**a,b**) are congruent for 26/44 × 100 = 59% of the structural subelements.

Overall, one can assume that both approaches complete each other, one recovering history using phylogenetic methods, and the other using principles from ecology and embryology for historical reconstruction. Accretion ranks of the 16S rRNA secondary structure subcomponents according to cladistic- and structure-based methods differ (Fig. 1). This analysis shows some congruence between accretion ranks obtained by the two independent methods, for 26 among 44 secondary structure elements (59%), which is not significantly more than 50% according to a one tailed sign test. The highest percentage of secondary structure subelements with reasonable match between accretion ranks from the two methods is for 16S rRNA domain 3, the lowest percentage is for domain 2. Notably, domain 4, presumed most ancient and consisting of two secondary structure subelements, has one element where both methods are highly congruent, and have very different ranks for the other subelement.

## Secondary structure classification

The overall impression resulting from Fig. 1 is that both structural and phylogenetic methods have some level of congruence, for a bit more than half of the secondary structure subelements, across all four 16S rRNA structural domains. Hence, for almost half of the secondary structure subelements, we do not know the accretion rank. A third independent method for estimating RNA history could improve the resolution of rRNA accretion ranks.

A method clustering RNA secondary structures found two main RNA secondary structure groups, one characterized by small, presumably ancient tRNA-like secondary structures, and a presumed more derived group, characterized by larger, rRNA-like secondary structures, including viruses^91,92^. The tRNA-like cluster was designed as tRNA-like because it included tRNAs. The decision to assume it is most ancient was not only based on the inclusion of tRNAs in that cluster. This cluster includes a high diversity of RNA types (viroids, ribozymes, tRNAs, replication origins, 5S rRNAs). Ancient groups tend to be more diverse because more time is available for “evolutionary radiation” (this term from species evolution might not be adequate in context of RNA species). The same rationale was applied to functional tRNA species, ranking as most ancient those with the highest diversity of isoacceptor tRNAs^93^.

The decision to consider the other RNA cluster as rRNA-like was because this cluster included all subdomains of small and large rRNAs. Note that this clustering is phenotypic, based on secondary structure similarities, not phylogenetic. The assumption that tRNA-like structures are primitive, and that rRNA-like structures are more derived is in line with the tRNA-accretion hypothesis for rRNA formation. Results show that tRNA-like RNAs have few unpaired nucleotides within stems (bulges); for rRNA-like secondary structures, the proportion of bulges among all unpaired nucleotides is greater. Bulges are targets for regulation and enzymatic degradation, properties of advanced metabolism. In prebiotic conditions, these might be disadvantageous, increasing degradation risks.

## Polarity of the tRNA-rRNA axis of RNA secondary structure evolution

This assumption about the evolutionary direction of secondary structures was tested explicitly on tRNAs from diverse organisms (organelles, Archaea, Bacteria, Eukaryota and Megavirales). First, similarities of all tRNAs from specific organisms with tRNA-like vs rRNA-like groups^91^ were estimated, projecting each tRNA secondary structure on a presumed tRNA-rRNA axis of RNA secondary structure evolution. Then correlations were calculated between the genetic code inclusion rank of the tRNA cognate amino acids^94^ and this tRNA-rRNA similarity score, expecting that tRNAs with relatively recent cognates have more rRNA-like secondary structures, and those with ancient cognates, are more typically tRNA-like. Results were overall positive (weakest in Eukaryota), confirming tRNA-rRNA polarity: two independent scales of evolutionary ranks, one for amino acids, and one for RNA secondary structures, converge^56^. Here again, polarity is not deduced from phylogenetic reconstructions, but from presumed orders of integration of the tRNA’s cognate amino acid.

Note that the phylogenetic and the structural methods also make polarity assumptions. In the former, these are deduced from cladistic parsimony principles^95^, in the latter, from structure: the more peripheral a structural element in the ribosome, the more recent, including information on stacking interactions among subdomains^78,96^. These results strengthen the hypothesis that tRNAs are ancestral and rRNAs derived.

## Independent references for RNA evolution

The tRNA-rRNA evolutionary axis score is based on a sample of known RNA secondary structures. Hence, it suffers from sampling biases, and from some level of circularity: biological data are used to infer on biological phenomena, a caveat it shares with the phylogenetic method. A possible solution to this is to use as reference theoretical minimal RNA rings, a set of short sequences designed *in silico* according to few basic constraints: the shortest possible sequence coding for a start and a stop codon, and once for each of the 20 biogenic amino acids.

These constraints define at most 25 circular RNA sequences of 22 nucleotides, which code according to partially overlapping codons, along three consecutive translation rounds, for a start codon, 20 different amino acids, and a stop codon. The stop codon is physically next to the start codon, closing the RNA ring. These RNA rings, mainly defined by coding sequences, resemble ancestral tRNAs^97,98^, with a predicted anticodon and its corresponding cognate amino acid for each RNA ring^55^.

The theoretical minimal RNA rings realistically mimic primitive RNAs and their evolution, along several coding properties^99–102^ and primary and secondary structure properties^50,56,57^. These properties coevolve with the genetic code integration order of the cognate amino acid matching the anticodon defined by homology of the RNA rings with ancestral tRNAs^50,56,57,99–102^. Considering that the design of RNA rings is purely rational and mainly based on the structure of the genetic code, this means that the genetic code’s structure intrinsically embeds information on the evolution of these various properties. However, we do not yet understand what determines these complex evolutionary trajectories.

Notably, the tRNA-rRNA scores obtained for secondary structures of these RNA rings, correlate, as observed for real tRNAs^56^, with the evolutionary ranks of integration of the cognate amino acids matching their predicted anticodons^57^. This parallels the result described in the previous section for regular tRNAs and the genetic code integration order of their cognate amino acid^56^. Here too, the polarity results from this order, not from phylogenetic reconstruction.

## Working hypothesis and predictions

Hence, RNA rings are designed as proto-mRNAs but have also properties that are expected for proto-tRNAs. As plausible proto-tRNAs, they are used here as references for ancestral RNAs, in line with results of evolutionary analyses of their different properties^50,56,57,99–102^. Analyses use similarities between RNA ring secondary structures and those of structural subelements of 16S rRNAs. The method assumes that high similarities with RNA ring secondary structures indicate ancient structural subelements, and low similarities recent 16S rRNA structural subelements. These similarities are then compared with accretion ranks produced by each of the phylogenetic and the structural hypotheses, expecting: 1. negative correlations if the different methods are producing congruent accretion ranks; 2. these correlations should be most negative for RNA rings with ancient cognate amino acids, and gradually be more positive for RNA rings with recent cognate amino acids.

## Materials and methods

The quantification of similarities between secondary structures is identical to previous analyses^56,57,91,92^. Optimal secondary structures of spliced RNA rings were predicted by Mfold^103^.

**Figure 2:**
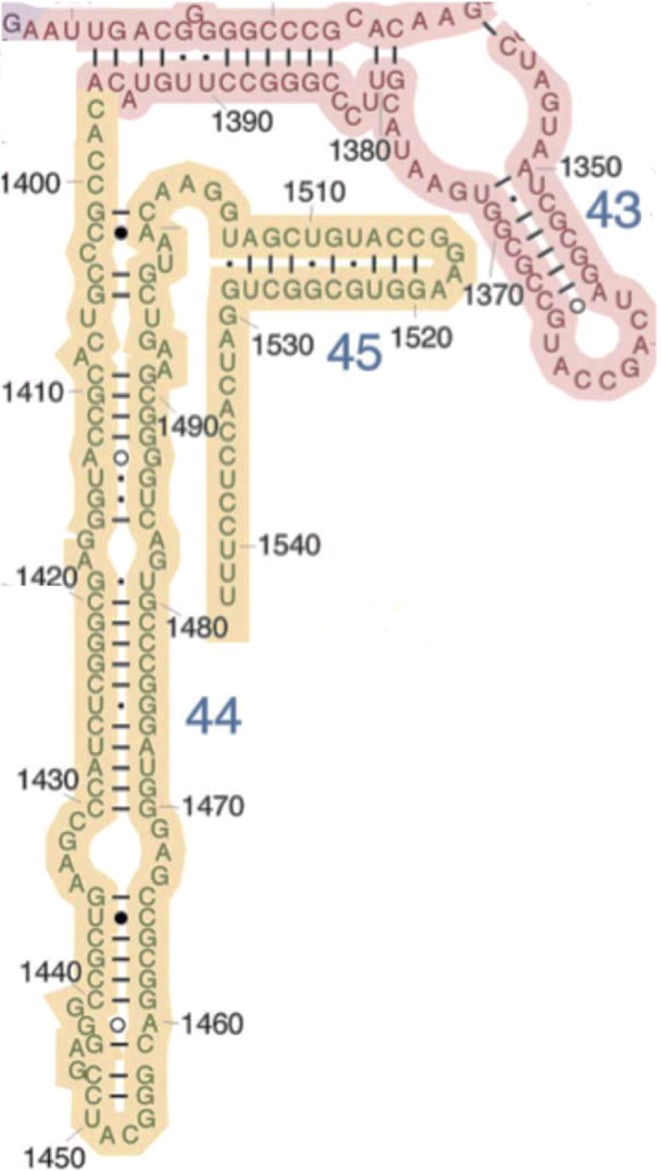
Secondary structure of domain IV (ochre, structural subelements h44 and h45) and part of domain III (pink, structural subelement h43) of 16S rRNA of *Thermus thermophilus* (adapted from http://rna.ucsc.edu/rnacenter/images/figs/thermus_16s_2ndry.jpg). Boundaries between secondary structure subelements are from Fig. 2 in^59^. Subelement h44 ranges from nucleotides 1397 to 1505. Its only external loop is from nucleotides 1450 to 1454. Sixty nucleotides are involved in stems (G-U included, C-A, U-C and G-A excluded and considered as internal bulges). Hence, a total of 41 nucleotides are considered unpaired, including the external loop. %stem = 100 × 60/101 = 59.4; %eloop = 100 × 4/41 = 9.8; %GCstem = 100 × 52/60 = 86.7; and %GCloops = 100 × 22/41 = 53.7.

Four secondary structure properties are extracted from secondary structures, as shown as example for structural subelement h45 from the archaean *Thermus thermophilus* 16S rRNA (Fig. 2): 1. the percentage of nucleotides in stems formed by complementary self-hybridization among nucleotides, %stem among all nucleotides in the sequence; 2. the percentage of nucleotides, among those in loops, that are in loops topping stems (external loops), as opposed to unpaired nucleotides forming bulges within stems (internal loops), %eloops; and the 3. stem and 4. loop GC contents, in percentages.

**Figure 3:**
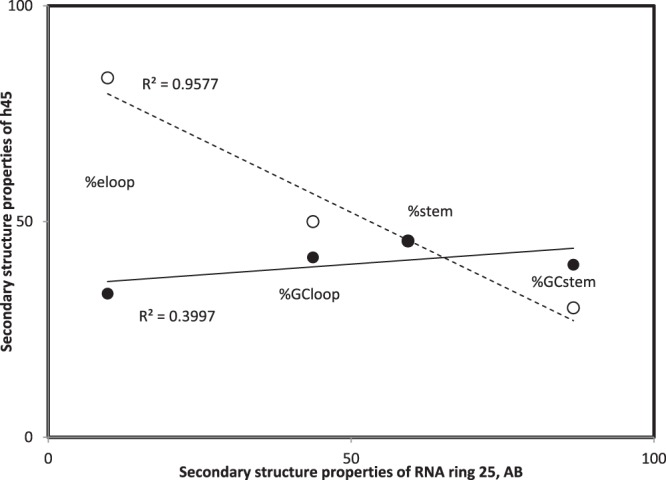
Similarity between secondary structure properties of structural subelement h45 of *Thermus thermophilus* 16S rRNA secondary structure and those of the secondary structure formed by AB (Table 1, secondary structures corresponding to splicing 7 and 19, filled and hollow symbols, respectively), as estimated by Pearson’s correlation coefficient r (note that r-squares are indicated in the figure). Each datapoint represents one of the four variables extracted from secondary structures, Y-axis values are from Fig. 2. Similarity with AB secondary structures, splicings 7 and 19, are: r = 0.633 and r = −0.979. The latter similarity is statistically significant at P < 0.05 (and indicates a stronger than random lack of similarity), the former indicates no similarity.

Similarities between two secondary structure pairs are estimated by Pearson correlation coefficients r between these four variables as obtained for each secondary structure (Fig. 3), in this case between values from Fig. 2 and those of secondary structures formed by two alternative splicings of RNA ring 25, also called AB^53^. Table 1 presents the four secondary structure variables for AB for all 22 alternative splicings of that RNA ring. Such data were obtained for all 25 RNA rings. Similar secondary structure data for 22 alternative splicings of RNA ring 13, called AL, were presented previously^57^, (therein Table 3). For each comparison, Fig. 3 has four datapoints for each secondary structure, one datapoint per secondary structure variable. For each datapoint, the X-axis is defined by the value obtained for the AB secondary structure, and the Y-axis by the value obtained for the corresponding variable for the 16S secondary structure subelement shown in Fig. 2. These pairings are not arbitrary: the x- and y-axis values are for the same secondary structure property, but for a different secondary structure (x-axis, RNA ring 25; y-axis, rRNA structural subelement, in this case h45 of *Thermus thermophilus*). Similarities are estimated by r, the more positive r, the more similar the secondary structures.

**Table 1:** Secondary structure variables extracted as in Fig. 2 explanations, for the 22 secondary structures formed by RNA ring 25 (AB, TATGAATGGTGCCATTCAAGACTA)^53^, according to 22 splicing positions. Splicing at position 1 corresponds to the splicing position producing highest homology with an ancestral tRNA^55^, each splicing of the RNA ring is shifted by a single nucleotide. These secondary structure data were used previously^57,109^^,^^110,111^^,^.

	%stem	%eloop	%GCstem	%GCloop
1	54.5	40.0	33.3	50.0
2	54.5	40.0	33.3	50.0
3	54.5	40.0	33.3	50.0
4	54.5	40.0	33.3	50.0
5	54.5	40.0	33.3	50.0
6	54.5	40.0	33.3	50.0
7	45.5	33.3	40.0	41.7
8	36.4	28.6	25.0	50.0
9	36.4	28.6	37.5	42.9
10	36.4	28.6	37.5	42.9
11	45.5	50.0	20.0	58.3
12	54.5	60.0	33.3	50.0
13	63.6	75.0	42.9	37.5
14	72.7	100.0	43.8	33.3
15	63.6	75.0	42.9	37.5
16	54.5	60.0	33.3	50.0
17	45.5	50.0	20.0	58.3
18	36.4	42.9	25.0	50.0
19	45.5	83.3	30.0	50.0
20	45.5	83.3	30.0	13.3
21	45.5	33.3	40.0	41.7
22	54.5	40.0	33.3	50.0

The secondary structure variables of all secondary structure subelements of two Archaea, *Thermus thermophilus* and *Sulfolobus solfataricus*^104^ (Table 2), two bacteria, *Escherichia coli* and *Streptomyces coelicolor*^105^ (Table 3), and the 18S rRNA of two eukaryotes, *Homo sapiens* and *Saccharomyces cerevisiae* (Table 4). Secondary rRNA structures for prokaryote 16S of *Thermus thermophilus*, *Escherichia coli*, and eukaryote 18S *Saccharomyces cerevisiae* and *Homo sapiens* are available at http://apollo.chemistry.gatech.edu/RibosomeGallery/.

**Table 2:** Variables extracted from secondary structures of 16S rRNA of archaeans *Thermus thermophilus* and *Sulfolobus solfataricus*. Columns are: 1. secondary structure subelement of 16S rRNA; 2 and 3. accretion ranks according to phylogenetic and structural models, respectively^59,78^ and 4–7, secondary structure variables as in Table 1 (explained in Figs. 2 and 3). Domains range from 1. h1-h18; 2. h19-h27; 3. h28-h43; 4. h44-h45.

#	Phyl	Str	Thermus thermophilus				Sulfolobus solfataricus			
			%ste	#eloo	%GCst	%GClo	%ste	#eloo	%GCst	%GClo
h1	25	3.3	43.5	30.8	60.0	30.8	19.0	0.0	50.0	58.8
h2	29	4	75.0	0.0	133.3	0.0	66.7	0.0	50.0	0.0
h3	10	4	62.5	0.0	60.0	50.0	71.4	0.0	75.0	25.0
h4		7	94.1	0.0	93.8	0.0	100.0		88.9	
h5	20	9	54.5	0.0	58.3	40.0	54.5	0.0	83.3	40.0
h6	10	12	72.7	25.0	81.3	58.3	62.1	54.5	61.1	45.5
h6a		12	100.0		66.7		0.0	0.0		87.5
h7		15	81.0	0.0	79.4	25.0	68.1	0.0	81.3	20.0
h8	30	18	58.8	28.6	95.0	35.7	74.3	44.4	80.8	11.1
h9	21	18	55.2	30.8	81.3	38.5	72.2	60.0	57.7	30.0
h10	37	15	66.7	75.0	100.0	25.0	69.0	88.9	55.0	44.4
h11	2	19	63.6	37.5	53.6	56.3	58.3	35.0	78.6	35.0
h12	17	12	72.7	66.7	75.0	66.7	71.0	44.4	77.3	33.3
h13	24	17	43.5	92.3	100.0	53.8	58.3	60.0	71.4	50.0
h14	28	17	53.3	57.1	75.0	57.1	61.5	80.0	100.0	60.0
h15	16	7	52.9	25.0	77.8	31.3	58.8	28.6	90.0	28.6
h16	22	26	48.5	23.5	81.3	29.4	66.7	66.7	83.3	50.0
h17	12	26	36.0	12.5	100.0	37.5	64.5	63.6	70.0	9.1
h18	33	7	51.1	17.4	91.7	56.5	54.2	63.6	69.2	72.7
h19	22	4	0.0	0.0		23.1	0.0	0.0		33.3
h20	18	7	63.6	0.0	92.9	50.0	76.9	0.0	75.0	50.0
h21	27	20	56.5	25.0	134.6	40.0	73.8	29.4	81.3	23.5
h22	11	20	60.0	0.0	140.0	70.0	66.7	0.0	100.0	58.3
h23	7	20	48.9	26.1	72.7	34.8	62.2	35.3	67.9	35.3
h23a	38	24	14.3	33.3	100.0	58.3	20.0	100.0	100.0	68.8
h24	8	5	51.1	39.1	87.5	30.4	54.2	40.9	88.5	27.3
h25	23	3	72.0	0.0	100.0	42.9	77.8	0.0	85.7	50.0
h26a	39	21	25.0	33.3	100.0	25.0	26.7	36.4	100.0	45.5
h26	9	21	80.0	60.0	70.0	20.0	88.2	75.0	63.3	0.0
h27	19	3	45.2	23.5	85.7	35.3	69.2	50.0	55.6	37.5
h28	9	3	73.7	0.0	71.4	50.0	83.3	0.0	70.0	25.0
h29	36	6	83.3	0.0	70.0	0.0	85.7	0.0	58.3	50.0
h30	15	8	78.6	0.0	59.1	66.7	74.1	0.0	75.0	57.1
h31	34	10	28.6	40.0	50.0	35.0	14.3	33.3	100.0	33.3
h32	19	10	77.8	0.0	85.7	25.0	82.4	0.0	92.9	33.3
h33	20	11	66.7	0.0	62.5	37.5	55.3	33.3	76.9	38.1
h33a		27	68.6	72.7	87.5	36.4				
h34	3	11	63.6	0.0	82.1	37.5	73.2	0.0	80.0	72.7
h35	35	22	80.0	0.0	66.7	66.7	83.3	0.0	60.0	133.3
h36	26	22	42.9	100.0	166.7	50.0	42.9	100.0	33.3	50.0
h37	31	23	44.4	60.0	87.5	20.0	44.4	60.0	100.0	30.0
h38		22	69.0	0.0	40.0	44.4	89.5	0.0	70.6	50.0
h39	32	22	55.6	62.5	100.0	62.5	77.8	100.0	57.1	100.0
h40	21	25	51.9	30.8	92.9	30.8	56.0	36.4	92.9	45.5
h41		14	51.9	0.0	78.6	23.1	55.2	0.0	93.8	30.8
h41a	14	14	45.7	36.8	100.0	31.6	75.0	50.0	66.7	25.0
h42	17	16	40.0	33.3	75.0	54.2	48.8	38.1	75.0	52.4
h43	18	6	40.0	47.6	78.6	33.3	47.4	25.0	77.8	45.0
h44	1	1	58.3	9.3	93.3	48.8	55.8	10.9	69.0	54.3
h45	13	2	54.1	23.5	65.0	47.1	58.8	28.6	5.0	50.0

**Table 3:** Variables extracted from secondary structures of 16S rRNA of bacteria *Escherichia coli* and *Streptomyces coelicolor*. Columns 2–9 correspond to columns 4–11 in Table 2.

#	*Escherichia coli*				Streptomyces coelicolor			
	%ste	#eloo	%GCst	%GClo	%ste	#eloo	%GCst	%GClo
h1	63.6	62.5	42.9	25.0	48.9	33.3	52.2	41.7
h2	72.7	50.0	43.8	33.3	60.0	0.0	66.7	0.0
h3	54.5	50.0	50.0	30.0	69.0	0.0	60.0	44.4
h4	45.5	25.0	30.0	42.9	88.9	0.0	87.5	50.0
h5	72.7	50.0	43.8	16.7	66.7	0.0	66.7	16.7
h6	63.6	37.5	35.7	37.5	61.1	28.6	59.1	50.0
h6a	54.5	30.0	41.7	30.0	0.0	0.0		66.7
h7	63.6	62.5	42.9	37.5	69.6	0.0	71.9	28.6
h8	45.5	41.7	30.0	50.0	62.5	33.3	65.0	58.3
h9	72.7	50.0	31.3	66.7	62.9	30.8	68.2	30.8
h10	72.7	50.0	56.3	0.0	44.4	80.0	100.0	80.0
h11	54.5	50.0	58.3	20.0	60.0	35.0	60.0	45.0
h12	45.5	41.7	40.0	41.7	59.3	63.6	93.8	36.4
h13	63.6	37.5	50.0	25.0	43.5	92.3	80.0	53.8
h14	63.6	37.5	50.0	25.0	57.1	66.7	50.0	50.0
h15	72.7	50.0	37.5	50.0	52.9	25.0	66.7	37.5
h16	45.5	41.7	30.0	50.0	51.6	26.7	75.0	46.7
h17	81.8	75.0	38.9	25.0	43.9	52.2	72.2	34.8
h18	81.8	75.0	38.9	25.0	51.1	69.6	70.8	60.9
h19	45.5	25.0	20.0	50.0	0.0	0.0		40.0
h20	63.6	37.5	50.0	12.5	55.2	0.0	81.3	38.5
h21	72.7	50.0	43.8	16.7	70.8	26.3	52.2	31.6
h22	36.4	21.4	50.0	28.6	80.0	0.0	44.4	66.7
h23	45.5	25.0	50.0	25.0	53.3	28.6	62.5	38.1
h23a	54.5	40.0	33.3	50.0	37.5	40.0	100.0	60.0
h24	54.5	50.0	58.3	20.0	52.2	40.9	79.2	31.8
h25	63.6	37.5	50.0	25.0	66.7	0.0	71.4	42.9
h26a	63.6	62.5	42.9	37.5	26.7	36.4	100.0	27.3
h26	54.5	30.0	25.0	60.0	76.2	93.3	58.3	26.7
h27	63.6	30.0	35.7	37.5	57.1	33.3	68.8	33.3
h28	54.5	30.0	41.7	30.0	85.7	0.0	75.0	50.0
h29	63.6	37.5	50.0	12.5	80.0	0.0	75.0	66.7
h30	63.6	62.5	42.9	37.5	71.4	0.0	65.0	50.0
h31	54.5	50.0	41.7	40.0	28.6	40.0	50.0	35.0
h32	72.7	50.0	31.3	66.7	82.4	0.0	64.3	0.0
h33	72.7	50.0	56.3	0.0	69.6	0.0	56.3	42.9
h33a	63.6	62.5	50.0	25.0	53.3	64.3	68.8	42.9
h34	54.5	50.0	50.0	30.0	75.6	0.0	67.6	27.3
h35	72.7	50.0	43.8	33.3	75.0	0.0	66.7	50.0
h36	72.7	50.0	56.3	0.0	54.5	80.0	50.0	60.0
h37	72.7	50.0	37.5	50.0	50.0	75.0	87.5	25.0
h38	27.3	18.8	83.3	25.0	80.0	0.0	70.8	33.3
h39	72.7	50.0	43.8	16.7	61.5	50.0	68.8	60.0
h40	81.8	75.0	38.9	25.0	42.9	25.0	75.0	56.3
h41	45.5	25.0	20.0	50.0	63.6	0.0	85.7	50.0
h41a	54.5	30.0	58.3	10.0	50.0	25.0	75.0	43.8
h42	81.8	75.0	38.9	25.0	39.0	32.0	75.0	44.0
h43	45.5	25.0	40.0	41.7	37.8	34.8	64.3	43.5
h44	27.3	18.8	66.7	25.0	59.8	11.6	79.7	39.5
h45	54.5	40.0	33.3	50.0	50.0	20.0	70.0	45.0

**Table 4:** Variables extracted from secondary structures of 16S rRNA of eukaryotes *Homo sapiens* and *Saccharomyces cerevisiae*. Columns 2–9 correspond to 4–11 in Table 2.

#	%ste	#eloo	%GCst	%GClo	%ste	#eloo	%GCst	%GClo
	Homo sapiens				Saccharomyces cerevisiae			
h1	52.6	66.7	70.0	44.4	50.0	70.0	70.0	30.0
h2	36.4	57.1	50.0	0.0	50.0	0.0	50.0	0.0
h3	66.7	0.0	25.0	60.0	84.6	0.0	27.3	25.0
h4	30.8	0.0	50.0	44.4	25.0	0.0	0.0	33.3
h5	57.1	0.0	66.7	22.2	54.5	0.0	83.3	10.0
h6	35.0	38.5	50.0	50.0	35.9	40.0	57.1	20.0
h6a	50.0	0.0	133.3	33.3	75.0	0.0	66.7	50.0
h7	50.0	0.0	45.0	45.0	35.7	0.0	30.0	25.0
h8	31.4	14.3	56.3	45.7	41.9	20.0	50.0	20.0
h9	53.8	13.3	84.3	58.3	54.1	20.5	34.8	25.6
h10	66.7	66.7	88.9	77.8	66.7	62.5	75.0	37.5
h11	50.0	34.6	53.8	57.7	54.9	30.4	57.1	39.1
h12	53.3	57.1	125.0	57.1	72.0	57.1	55.6	42.9
h13	43.5	76.9	80.0	46.2	38.5	62.5	80.0	37.5
h14	42.9	50.0	66.7	37.5	42.9	50.0	66.7	50.0
h15	54.8	35.7	70.6	64.3	50.0	31.3	56.3	43.8
h16	64.9	30.8	50.0	23.1	61.1	28.6	59.1	21.4
h17	35.3	50.0	33.3	36.4	27.8	42.3	50.0	26.9
h18	47.1	25.9	62.5	48.1	49.0	16.0	62.5	52.0
h19	0.0	0.0		12.5	0.0	0.0		7.1
h20	72.7	0.0	37.5	50.0	60.9	0.0	35.7	44.4
h21	61.7	29.3	69.7	52.4	49.3	30.4	58.0	28.7
h22	59.5	0.0	68.2	33.3	57.1	0.0	70.8	38.9
h23	55.8	31.6	70.8	42.1	57.8	31.6	30.8	26.3
h23a	25.0	33.3	100.0	41.7	25.0	33.3	100.0	25.0
h24	45.3	31.0	66.7	31.0	46.2	32.1	50.0	32.1
h25	60.0	0.0	75.0	37.5	63.6	0.0	64.3	25.0
h26a	40.0	44.4	66.7	33.3	34.0	54.8	81.3	25.8
h26	76.9	44.4	70.0	77.8	40.0	44.4	33.3	22.2
h27	48.5	23.5	50.0	35.3	62.9	30.8	63.6	46.2
h28	80.0	0.0	53.6	100.0	75.7	0.0	53.6	100.0
h29	62.5	0.0	50.0	83.3	85.7	0.0	41.7	50.0
h30	69.2	0.0	83.3	75.0	71.4	0.0	75.0	75.0
h31	33.3	40.0	50.0	30.0	33.3	40.0	50.0	40.0
h32	66.7	0.0	100.0	33.3	85.7	0.0	75.0	0.0
h33	40.0	33.3	60.0	33.3	40.0	33.3	50.0	0.0
h33a								
h34	63.8	0.0	60.0	105.9	66.7	0.0	56.3	56.3
h35	66.7	0.0	40.0	80.0	66.7	0.0	60.0	120.0
h36	60.0	100.0	33.3	75.0	60.0	100.0	33.3	50.0
h37	38.5	75.0	140.0	0.0	40.0	50.0	62.5	8.3
h38	66.7	0.0	62.5	50.0	84.2	0.0	81.3	0.0
h39	63.2	19.0	50.0	76.2	71.4	33.3	53.3	8.3
h40	55.2	38.5	75.0	23.1	69.0	33.3	55.0	33.3
h41	37.5	0.0	61.1	70.0	56.4	0.0	54.5	58.8
h41a	82.4	66.7	46.4	66.7	51.4	23.5	72.2	17.6
h42	37.8	43.5	78.6	30.4	42.1	45.5	56.3	27.3
h43	72.7	55.6	50.0	33.3	55.2	53.8	56.3	38.5
h44	61.9	9.4	69.8	45.3	63.8	7.8	55.6	37.3
h45	62.5	33.3	60.0	25.0	62.5	33.3	60.0	25.0

### Step by step description of analyses

1. There are 25 RNA rings, each 22 nucleotide long. These are considered according to the splicing matching homology with ancestral tRNAs, as shown previously (Table 1 in^50,57,100,102^ and Table 2 in^101^).
2. Each RNA ring can be spliced at 22 positions, and a different optimal secondary structure (predicted by Mfold^103^) exists for RNA ring sequences spliced at each potential splicing position. The 25 RNA rings form 25 × 22 = 550 secondary structures.
3. Four secondary structure variables are extracted from each of these 550 secondary structures. Table 1 presents as an example these four variables for the 22 alternative splicings of a specific RNA ring, RNA ring 25.
4. For each of the (about 45) structural subelements of small rRNA subunits of the 6 examined organisms, the four secondary structure variables are extracted, as was done for the 550 RNA ring structures at step 3. These variables are presented for the 6 × 45 = 270 secondary structure subelements presented in Tables 2–4.
5. The secondary structures of RNA rings are compared to the secondary structures of rRNA structural subelements by analyses as presented in Fig. 3. These analyses plot the values obtained for each of the 4 secondary structure variables of a rRNA structural subelement as a function of the corresponding values obtained for a given RNA ring secondary structure. A Pearson correlation coefficient r, called rS, estimates similarities between rRNA and RNA ring secondary structures. Figure 3 presents comparisons between 16S rRNA subelement h45 of *Thermus thermophilus* and two RNA ring 25 secondary structures, one obtained by splicing that ring at position 7, and one at position 19.
  For each of the 550 RNA ring secondary structures, there are as many rS as there are rRNA secondary structure subelements, about 45.
6. According to our hypothesis, the (about) 45 rSs comparing a given RNA ring secondary structure to all rRNA structural subelements are potential estimates of the accretion order of the rRNA secondary structures.
7. These rSs are compared to the accretion order of the rRNA secondary structure subelements, as these were determined by other methods and published by other authors (separately for each cladistic and structural accretion ranks). This comparison is done by calculating the Pearson correlation coefficient between the rS and the accretion orders, producing rH, one for the cladistic method, rHphyl, and one for the structural method, rHstru. Note that rS are z-transformed before calculating rH using the formula z = −ln((1 + r)/(1 − r)). The z transformation linearizes the scale of r, which is not linear.
8. Hence, each of the 550 RNA ring secondary structures produces one rHphyl and one rHstru per organism. For each organism, there are 550 rHphyls and 550 rHstrus. The minimal and maximal rHphyls and rHstrus for each organism are in Table 5. Table 5 includes percentages of negative rHphyls and rHstrus (the working hypothesis expects negative rHs), and numbers of negative and positive rHphyls and rHstrus that have two tailed P < 0.05.

**Table 5:** Most negative and most positive Pearson correlations coefficients r (x100) (rH) between accretion ranks according to phylogenetic (rHphyl) and structural (rHstru) models with secondary structure similarities with RNA rings for 16S rRNAs of six organisms, and percentages of rHs (%neg) that are negative as expected by the working hypothesis among the 550 correlation calculated for each rHphyl and rHstru, for each organism. * indicates statistically significant differences (P < 0.05) from 50% (550/2 = 275 negative rHphyl and rHstru are expected if the sign of rH has an unbiased distribution between negative and positive trends) according to a chi-square test. “Co” indicates the cognate amino acid corresponding to the anticodon of the RNA ring(s) producing these correlations. Cognate G always corresponds to RNA ring 25 (AB). N indicates numbers of datapoints involved in the calculation of rH correlation coefficients.

Taxon	Phyl,	rHphyl	Co	%neg	Max	Co	Stru,	rHstru	Co	%neg	Max	Co
	N	Min					N	Min				
**Archaea**												
*Thermus thermophilus*	38	−46.1	G	62.6*	44.2	Sec	48	−40.7	CDEM	67.9*	38.7	G
*Sulfolobus solfataricus*	44	−54.7	AKQSW	86.9*	37.4	G	46	−50.9	AGLNPQS	54*	51.2	Sec
**Bacteria**												
*Escherichia coli*	42	−37.4	G	64.7*	35.6	Sec	48	−35.6	ALNPQS	64.2*	35.0	Sec
*Streptomyces coelicolor*	39	−46.8	A	90.9*	30.7	R	48	−32.9	GLT	72*	32.3	G
**Eukaryota**												
*Homo sapiens*	43	−36.0	N, R	53.5	32.3	Sec	48	−25.5	AKQSW	80.4*	19.1	G
*Saccharomyces cerevisiae*	43	−36.1	S	79.1*	27.5	Sec	48	−36.0	GT	53.6	34.9	Sec
9. For any given RNA ring secondary structure, there are 6 rHphyls and 6 rHstrus, because analyses were done for 6 organisms. There are in total 6 × 550 = 3300 rHphyls and 3300 rHstrus. Further analyses describe general patterns within these data, according to RNA rings, and according to splicing positions.
10. For each RNA ring, there are 22 secondary structures which produce 22 rHphyls and 22 rHstrus per organism, hence 6 × 22 = 132 rHphyls and 132 rHstrus across all 6 organisms. An alternative way to explain this is: for each of the 25 RNA rings, there are 3300/22 = 132 rHphyls and 132 rHstrus across all 6 organisms.
  Percentages of negative rHphyls and rHstrus for each RNA ring (calculated among the 132 rHphyls and among the 132 rHstrus, pooling all organisms) are used in the y axis of Fig. 4.

**Figure 4:**
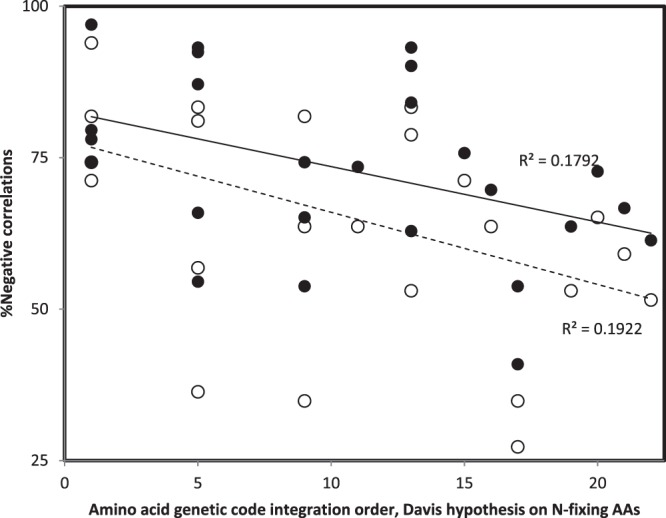
Percentage of negative Pearson correlation coefficients r between accretion ranks (phylogenetic method, filled symbols; structural method, hollow symbols) and similarities between 16S rRNA and RNA ring secondary structures, r’s pooled across organisms and secondary structures formed by the 22 alternative splicing of each RNA ring, as a function of the genetic code integration order of the RNA ring’s predicted cognate amino acid according to Davis’s hypothesis on N-fixing amino acids^105^. The working hypothesis expects negative r’s in particular for ancient amino acids.
11. There are 25 RNA rings. Hence, for a given splicing position, there are 25 rHphyls and 25 rHstrus. Pooling these data across 6 organisms, for any given splicing position, there are 6 × 25 = 150 rHphyls and 150 rHstrus across all 6 organisms. Percentages of negative rHphyls and rHstrus for each splicing position, calculated from these 150 rHphyls and 150 rHstrus, consist the y axis in Fig. 5.

**Figure 5:**
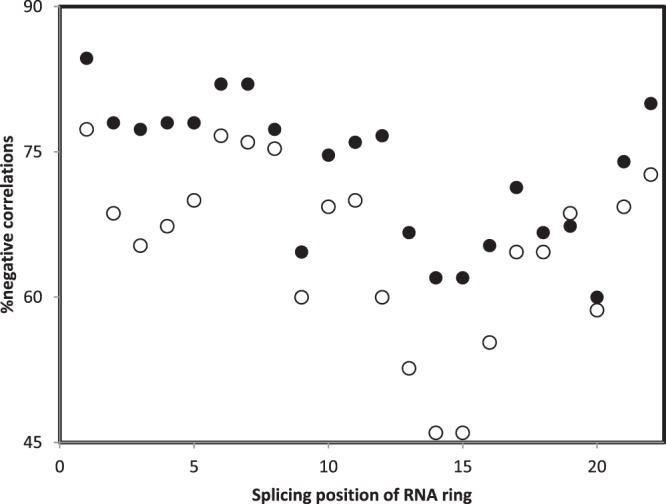
Percentage of negative Pearson correlation coefficient r between accretion ranks (phylogenetic method, filled symbols; structural method, hollow symbols) and similarities between 16S rRNA and RNA ring secondary structures, r’s pooled across organisms and RNA rings, as a function of the splicing position of the RNA ring. The splicing position with the highest percentage of negative correlations is position “1”, which corresponds to the splicing that produces the best homology between RNA rings and ancestral tRNAs^57^.

Analyses in Table 5, Figs. 4 and 5 each take into consideration all 3300 rHphyls and 3300 rHstrus. Hence, these are not biased representations of the data. They show separately effects of each ‘treatment factor’ (organism, RNA ring, splicing position) on each rHphyl and rHstru.

## Results and discussion

There are 25 theoretical minimal RNA rings. Each has exactly 22 nucleotides, hence each RNA ring has 22 alternative splicing positions. Different splicings produce different sequences forming different secondary structures, as shown for RNA ring 25, AB, in Table 1, and previously for RNA rings 9^106^, (therein Table 1) and 13^57^, (therein Table 3). Hence, there are 25 × 22 = 550 secondary structures to which secondary structure subelements of the 16S rRNAs can be compared.

The secondary structure variables shown in Table 1 and Figs. 2 and 3 were extracted for each of these 550 RNA ring secondary structures and are compared, as shown in Fig. 3, with the corresponding secondary structure variables of all secondary structure subelements of all six organisms considered here.

Table 5 shows the most negative and the most positive rH correlations between secondary structure similarities and accretion ranks according to the phylogenetic and the structural method for each of the six organisms (rHphyl and rHstru, respectively). Similarities (rS) were between the secondary structure variables described in Tables 2–4 and corresponding variables for the secondary structures formed by each of the 22 alternative splicings of each of the 25 theoretical minimal RNA rings. Considering that the main prediction of the working hypothesis expects negative correlations, it is notable that in each organism, the absolute values of the negative correlation is larger than the absolute value of the positive correlation, besides for one among 12 comparisons, according to the structural method, for *Sulfolobus solfataricus*.

Similarly, percentages of negative correlations are in all organisms, for both rHphyl and rHstru, always greater than 50%, significantly so according to a chi-square test in all but three among 12 tests, rHphyl in *Homo*, rHstru in yeast and in *Sulfolobus*. In addition, percentages of negative rHs are significantly greater for rHphyl than rHstru within three among six species, *Sulfolobus*, *Streptomyces* and yeast. In *Homo*, percentages of negative rHstru were significantly greater than percentages of negative rHphyl. The overall pattern is that results match the working hypothesis, and this more for rHphyl than rHstru. The opposite occurs in *Homo*. This could be interpreted as due to recent evolution of small rRNA structure in that species, but would require additional analyses and data from other species.

A second noteworthy point is that the most positive correlations are in 7 among 12 cases with RNA ring 2, which has a predicted anticodon for a stop codon, coding sometimes for selenocysteine. This is presumably one of the latest amino acids integrated in the genetic code (21^st^). This result fits the prediction that the most positive correlations between accretion ranks and secondary structure similarities would correspond to RNA rings with recent cognates. In other words, these secondary structures would not be references for initial RNAs starting the accretion process, but for the latest RNAs in the accretion process.

Figure 4 plots percentages of negative r’s between accretion ranks and secondary structure similarities between small rRNA subelements and RNA ring secondary structures, pooling all organisms and alternative splicings of RNA rings. Patterns confirm several points: 1. Most correlations between accretion ranks and secondary structure similarities are negative as expected by the working hypothesis, for most RNA rings; 2. In most cases, there are more negative correlations for the phylogenetic than the structural method for reconstructing accretion ranks; 3. Percentages of negative correlations decrease with the genetic code integration order of the cognate amino acid of RNA rings (see above comments for RNA ring with selenocysteine as predicted cognate).

Figure 5 presents the percentages of negative r’s between accretion ranks and secondary structure similarities between small rRNA subelements and RNA ring secondary structures, pooling all organisms and RNA rings, as a function of RNA ring splicing position. Results show that correlations are most frequently negative, meaning fitting the working hypothesis, when RNA rings are spliced at position “1”. This is the position defined by the highest homology between the RNA ring and an ancestral tRNA^55^. This observation is also in line with the working hypothesis that RNA rings are proto-tRNAs, and that accretion of proto-tRNAs, tRNAs and tRNA-like RNAs formed rRNAs. Note that the assumption that RNA rings are proto-tRNAs is under debate^107^. Nevertheless, and apparently confirming this status of proto-tRNAs, pseudo-phylogenetic analyses of RNA ring sequences reveal two clusters of RNA rings, one coinciding with RNA rings whose presumed cognate amino acid is the cognate of tRNAs for which the tRNA acceptor stem includes a primitive code^108^.

Particularly noteworthy is that results of analyses presented here for the small rRNA subunit are in line with results obtained for the large rRNA subunit^106^. These analyses compared structural subelements of the large rRNA subunit with the same RNA ring secondary structures as those used here. As described here for the small rRNA, for the large rRNA subunit, comparisons with RNA ring secondary structures show that: a. are slightly more congruent with the phylogenetic than the structural method; b. results are strongest for comparisons with RNA rings with predicted ancient cognate amino acids; c. weakest for comparisons with RNA rings with predicted recent cognate amino acids.

## Conclusions

Results are strong corroboration of the working hypothesis that tRNA accretions formed rRNAs. They show that RNA rings are likely proto-tRNAs, and that these are good reference points for primitive RNAs in general, and tRNAs in particular. Results confirm that RNA ring cognates are good estimates for RNA ring evolutionary ranks, and that similarities between secondary structures bear information on evolutionary direction of RNA secondary structures, from tRNA to rRNA-like, also among rRNA structural subelements. This has been suggested by several previous lines of analyses presented in the Introduction^10–12,15–21,56,57,91,92^, expanding upon evidences for common origins for tRNAs and rRNAs^1–4,7–9^. Analyses presented here for the small rRNA subunit show greater congruence between accretion orders derived from the secondary structure method used here and the phylogenetic method than between the former and the structural method. Similar analyses done for the large rRNA subunit produce qualitatively similar results, independently confirming our method and evolutionary conclusions. Overall, both phylogenetic and structural methods produce accretion orders that are congruent with the secondary structure method applied through the tRNA-rRNA axis of RNA secondary structure evolution. It is probable that the structural methods are more prone to errors due to evolutionary convergences than the phylogenetic method, though convergences remain the main difficulty in reconstructing evolution.
